# Small airway dysfunction across alpha-1 antitrypsin deficiency genotypes with preserved spirometry

**DOI:** 10.1007/s00421-026-06278-7

**Published:** 2026-05-29

**Authors:** Alessandra Marchese, Annalisa Frizzelli, Rocco Accogli, Roberta Pisi, Olha Bondarenko, Alfredo Chetta, Marina Aiello

**Affiliations:** Pneumology, Department of Medicine and Surgery, University Hospital of Parma, Via Gramsci 14, Parma, 43126 Italy

**Keywords:** Alpha-1 antitrypsin deficiency, Small airways dysfunction, Spirometry, Impulse oscillometry system

## Abstract

**Purpose:**

Alpha-1 antitrypsin deficiency (AATD) is a genetic disorder that generally predisposes to pulmonary emphysema with or without chronic obstructive pulmonary disease (COPD). Despite the lack of spirometric obstruction, early Small Airways Dysfunction (SAD) may occur in AATD patients preceding lung damage.

**Methods:**

We conducted a retrospective, single-center study on 60 AATD patients without airflow obstruction (FEV₁/FVC ≥ 0.70). SAD was defined as: reduced spirometric flows (≥2 of FEF25–75, FEF50, FEF75 <65% predicted) and/or IOS R5–R20 value greater than 0.07 kPa•s•L⁻¹. Correlation and agreement between spirometry and IOS were assessed, as well as associations with Modified Medical Research Council (mMRC) dyspnea scores.

**Results:**

SAD was detected in 23% of patients by spirometry, 10% by IOS, and 13% by both methods. A slight agreement was found between spirometric and IOS criteria (κ = 0.22). Only IOS-defined SAD was significantly associated with dyspnea (mMRC ≥2; p = 0.05), and R5–R20 values correlated with mMRC scores (r = 0.38, p = 0.003). No association was observed between spirometry-defined SAD and symptoms.

**Conclusions:**

These findings support IOS as a partially complementary tool for the early detection of peripheral airway impairment in AATD. Moreover, IOS but not spirometry criteria were related to breathlessness perception in AATD patients without airflow obstruction, with IOS proving to be a clinically more sensitive measure.

**Supplementary Information:**

The online version contains supplementary material available at 10.1007/s00421-026-06278-7.

## Introduction

Alpha-1 antitrypsin deficiency (AATD) is an underdiagnosed genetic disorder with an autosomal co-dominant inheritance pattern (Stoller and Aboussouan 2012). It results from polymorphisms in the *SERPINA1* gene (chr.14). A marked reduction or complete absence of circulating AAT predisposes individuals to liver and lungs diseases (Strnad et al. 2020). In the lung, the most common clinical manifestations are emphysema, asthma and bronchiectasis (Stoller and Aboussouan 2012, Strnad et al. 2020). Given the heterogeneity of SERPINA1 genotypes, AATD encompasses a broad spectrum of biochemical deficiency levels, from severe forms (ZZ, SZ, null, rare genotypes) to heterozygous variants (MZ, MS, M-like etc.). The functional implications of these milder genotypes remain poorly characterized (Ferrarotti et al. 2025). 

Small airways (defined as bronchioles < 2 mm in diameter) are essential for the transport and exchange of oxygen and carbon dioxide and at the same time they play a relevant role in lung mechanics, contributing to the subdivision of lung volumes (Frizzelli et al. 2022). Indeed, small airways determine the residual volume (RV) due to their tendency to collapse during maximal expiration (Frizzelli et al. 2022). It is of note that though small airways are an early site of airway injury, they can undergo extensive damage before symptoms appear, since they are considered as a “silent zone” due to their low resistance (Frizzelli et al. 2022).

Up to now, scarce evidence has been reported about small airways dysfunction (SAD) in AATD patients with preserved spirometry (Ferrari da Cruz et al. 2021, Stockley et al. 2017). Ferrari da Cruz et al. (Ferrari da Cruz et al. 2021) reported three AATD patients with SAD, assessed by single breath nitrogen washout but spirometry in the normal range, suggesting the relevance of the small airway function’s measurements in AATD. Stockley et al. (Stockley et al. 2017) in a large cohort of AATD patients showed that a reduction in forced expiratory flow between 25 and 75% (FEF_25−75_) of the forced vital capacity (FVC) was an early feature of lung disease in AATD and was associated with health status impairment. Accordingly, the functional assessment of small airways is relevant for the management of patients with AATD, although no universally accepted gold standard currently exists (Frizzelli et al. 2022).

It is worth noting that impulse oscillometry system (IOS) is a promising technique to assess peripheral airway function. IOS applies pressure to airways at a range of frequencies to measure components of respiratory impedance, including resistance and reactance (Frizzelli et al. 2022). Compared to spirometry, IOS does not require forced expiratory maneuvers and is less influenced by patient cooperation and lung elastic recoil. This allows IOS to potentially detect early peripheral airway abnormalities that may not yet be identified by conventional spirometry indices. However, IOS is less widely available and lacks universally standardized reference values, which may limit its routine clinical application (Liwsrisakun et al. 2025).

On the other hand, in a large cohort of subjects, Xiao et al. (2020) assessed SAD using three indicators of spirometry, namely FEF_25−75_, forced expiratory flow at 50% (FEF_50_), and at 75% (FEF_75_) of FVC; SAD was diagnosed when at least two of these three indicators were less than 65% of predicted values.

The primary aim of our study is to determine SAD using IOS and the three indicators of spirometry proposed by Xiao et al. (2020) in AATD patients and their relatives with preserved spirometry. We also assessed the relationship between spirometry and IOS indicators and clinical features of AATD. Our secondary objective was to assess the agreement between spirometry and IOS in detecting SAD in our cohort of subjects.

## Materials and methods

### Study population

This study was approved by the Ethics Committee “Area Vasta Emilia Nord (AVEN)”, approval number 27,533; dated July 13, 2018 in accordance with the Helsinki Declaration of 1964. The written consent was obtained from all participants.

This is a retrospective, observational, single-center study conducted at the regional referral outpatient clinic for the study and treatment of individuals with AADT of the University Hospital of Parma. Data were collected from July 2018 to October 2025. Patients were eligible if they had a confirmed AATD diagnosis (serum AAT levels < 113 mg/dL (20.8µmol/L) with molecular evidence of a pathogenic genotype (Ferrarotti et al. 2012), and no evidence of airflow obstruction, defined as forced expiratory volume at 1st second (FEV1)/FVC ≥ 0.70. Exclusion criteria included a diagnosis of AATD-related COPD and incomplete lung function data. We also recruited patient’s relatives in accordance to European Respiratory Society statement: diagnosis and treatment of pulmonary disease in α1-antitrypsin deficiency (Miravitlles et al. 2017).

In all individuals, in addition to demographic data (age, sex and BMI in kg/m^2^), smoking history and atopy, assessed by skin prick-tests, we collected information on respiratory diseases including emphysema, asthma and bronchiectasis. Serum AAT levels (mg/dL) were retrieved from laboratory reports and interpreted according to the local reference range (90–200 mg/dL). Inflammatory status was assessed by measuring C-reactive protein (CRP, mg/L), which was performed concomitantly with AAT quantification.

All individuals performed pulmonary function tests, including body plethysmography and IOS measurements.

### Lung function testing

Spirometry was conducted by using a flow-sensing spirometer connected to a computer for data analysis (Vmax 22 and 6,200; SensorMedics, Yorba Linda, CA, USA) adhering to established international guidelines (Graham et al. 2019). Among the recorded manoeuvres, the one yielding the highest combined value of FVC and FEV1 was chosen for analysis (Graham et al. 2019). Parameters such as FEV1, FVC, FEF_25–75_, FEF_50_, and FEF_75_ were measured and expressed both in litres and as percentages of predicted values (% pred) (Quanjer et al. 2012). Small airway dysfunction was identified when at least two out of the three flow parameters (FEF_25–75_, FEF_50_, FEF_75_) were found to be below 65% of their predicted values (FEF+) (Xiao et al. 2020).

IOS was performed by using the Jaeger MasterScreen-IOS (CareFusion Technologies; San Diego, CA, USA), following standard recommendations (Thamrin et al. 2020). To avoid any alterations in airway tone caused by forced expiratory efforts, IOS measurements were taken prior to performing spirometry. During the assessment, participants wore a nose clip and were seated in an upright position, breathing quietly through the mouthpiece. They were instructed to keep their neck slightly extended and to seal their lips firmly around the mouthpiece while supporting their cheeks with their hands to minimize artefacts. Each patient completed a minimum of three 30-second recordings, and mean values were calculated from these trials. SAD was defined based on the fall in resistance from 5 Hz to 20 Hz (R5–R20), using a conservative upper threshold of 0.070 kPa·s·L⁻¹, as suggested by reference standards (Lipworth et al. 2014, Oppenheimer et al. 2007, Cottini et al. 2023) (R5–R20+). Additionally, measurements of reactance at 5 Hz (X5, kPa·s·L⁻¹) and the reactance area (AX, kPa·L⁻¹) were included, as both are recognized indicators of peripheral airway impairment (Thamrin et al. 2020, Lipworth et al. 2014).

### Modified medical research council (mMRC)

The mMRC scale was used to assess the degree of dyspnoea. This test is based on five levels of increasing dyspnoea intensity, each corresponding to a specific situation. The scores allow for classification of symptom severity: a score of 0–1 indicates mild symptoms, while a score of ≥ 2 suggests severe symptoms. An mMRC score ≥ 2 is considered a significant risk factor (Fletcher 1960).

### Statistical analysis

A Shapiro-Wilk test was used to assess the distribution of the variables. Data were reported as mean ± standard deviation (SD) for the variables with normal distribution and as median [25th–75th percentile] for those with a non-normal distribution.

Statistical comparisons were performed using an unpaired t-test, Mann-Whitney test, or Pearson’s χ² test, as appropriate. Relationships between variables were assessed by Pearson correlation coefficient (r) or by Spearman’s rank correlation coefficient (ρ), depending on the data distribution. Effect size was interpreted according to Cohen’s conventions: values of r around 0.1 were considered small, around 0.3 medium, and greater than 0.5 large (Cohen 1988). Significant correlations were further assessed using linear regression analysis.

Interrater agreement was assessed using Cohen’s Kappa (κ) (Landis and Koch 1977). The κ values were categorized as follows: <0.00, “poor”; 0.00–0.20, “slight”; 0.21–0.40, “fair”; 0.41–0.60, “moderate”; 0.61–0.80, “substantial”; and 0.81–1.00, “almost perfect” agreement.

A p value < 0.05 was considered significant.

## Results

We recruited 60 AATD individuals, 31 patients affected by asthma and/or bronchiectasis (52%) and 29 relatives (48%), all without evidence of airflow obstruction (FEV_1_/FVC ≥ 0.70), who were consecutively referred to our outpatient clinic of University Hospital of Parma from July 2018 to October 2025.

The anthropometric, clinical, and functional characteristics as well as the genotypes distribution of subjects are summarized in Tables 1 and 2.

**Table 1:** Anthropometric, clinical and functional characteristics of 60 AATD subjects

	Total Cohort	Patients	Relatives	Patients Vs relatives
Anthropometric characteristics				
Age (years)	50 ± 16.0	51 ± 13	48 ± 19	*p* = 0.4
Sex, F/M	39/21	23/8	16/13	*p* = 0.12
BMI (kg/m²)	26 ± 5	27 ± 5	26 ± 6	*p* = 0.4
*Smoking habit*				
Current smokers [n(%)]	6 (10)	2(7)	4(14)	*p* = 0.41
Former smokers [n(%)]	14 (23)	6(19)	8(28)	
Never smokers [n(%)]	40 (67)	23(74)	17(58)	
Pack-years	12 ± 18	6 [4.5–17.5]	7 [1–20]	*p* = 0.7
*Comorbidity*				
Atopy yes/no	31/29	23/8	8/21	*p* = 0.0003
Asthma yes/no	24/36	24/36	/	/
Bronchiectasis yes/no	4/53	4/53	/	/
Asthma and Bronchiectasis yes/no	3/57	3/57	/	/
*Clinical characteristics*				
AAT (mg/dL)	86 [74.6–99.6]	88 [75–105]	82 [70–100]	*p* = 0.4
CRP (mg/L)	1 [0.5–1.9]	1[1–3]	1 [0.3-2]	*p* = 0.2
*Functional characteristics*				
FEV1,% pred	105 ± 18	102 ± 15	110 ± 19	*p* = 0.07
FVC, % pred	112.4 ± 2.3	110 ± 16	114 ± 20	*p* = 0.3
FEV1/FVC, % pred	98.4 ± 6.9	97 ± 6	99 ± 8	*p* = 0.13
FEF_25−75_, % pred	78.5 ± 30.3	72 ± 25	85 ± 34	*p* = 0.07
FEF_50_, % pred	82 ± 28	75 ± 23	89 ± 32	*p* = 0.02
FEF_75_, % pred	60.00 [45.00–86.00]	60 ± 29	78 ± 36	*p* = 0.03
TLC,% pred	106 ± 12	104 ± 10	109 ± 13	*p* = 0.12
R5-R20, kPa·s·L − 1	0.0300 [0.0100-0.0675]	0.0442 ± 0.0455	0.0500 ± 0.0979	*p* = 0.24
AX, kPa·s·L^− 1^	0.3300 [0.1500–0.6400]	0.3700 [0.2100–0.7000]	0.2000[0.1300–0.4500]	*p* = 0.02
X5, kPa·s·L^− 1^	-0.1300 [-0.1500- -0.0800]	-0.1300[-0.1600- -0.1000]	-0.1000[-0.1450- -0.0800]	*p* = 0.07

Statistically significant values are shown in bold (*p* < 0.05).

*BMI* body mass index; *FVC* forced vital capacity; FEV1, forced expiratory volume in 1 s; *FEF25-75* forced expiratory flow between 25 and 75%; *FEF50* forced expiratory flow at 50%; *FEF75* forced expiratory flow at 75%; *X5* 5 Hz reactance; *AX* reactance area.

**Table 2:** Genotypes Distribution among 60 AATD subjects

Genotypes [*n*(%)]	Total Cohort	Patients	Relatives
PI*MS	19 (31)	13(42)	6 (21)
PI*MZ	20 (32)	12(39)	8 (28)
PI*SS	3 (5)	2(6)	1 (3)
PI*SZ	2 (3)		2(7)
PI*MM_wurzburg_	1(2)	1(3)	
PI*M_procida_M_procida_	1(2)	1(3)	
PI*MM_whitstable_	1(2)	1(3)	
PI*MQ0_newmutation_	1(2)	1(3)	
PI*MQ0_ourèm_	3(5)		3(10)
PI*MM_malton_	2 (3)		2 (7)
PI*MP_lowell_	2 (3)		2 (7)
PI*ZY_orzinuovi_	1(2)		1 (3)
PI*MP_brescia_	1(2)		1 (3)
PI*MI	1(2)		1 (3)
PI*MQ0_clayton_	1(2)		1 (3)
PI*MQ0_parma_	1(2)		1 (3)

Twenty-four patients (77%) had a diagnosis of asthma assessed by metacholine challenge test, the mean provocative dose causing a 20% fall in FEV_1_ (PD20) ranging from 156.19 to 1500.80 mcg. Cylindrical bronchiectasis, assessed by chest Computed Tomography, was found in 4 patients (13%) and 3 patients (10%) had both asthma and bronchiectasis. None of the subjects met the criteria for COPD.

**Fig. 1:**
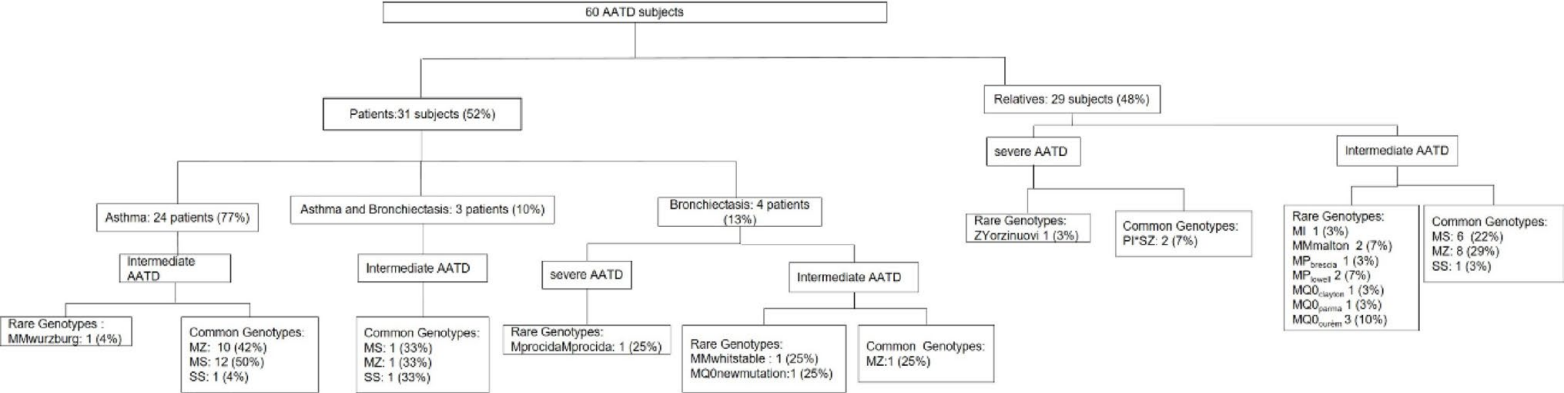
Genotype distribution on a cohort of 60 AATD individuals

Figure 1 shows a flow chart illustrating the cohort of 60 subjects with AATD.

**Fig. 2:**
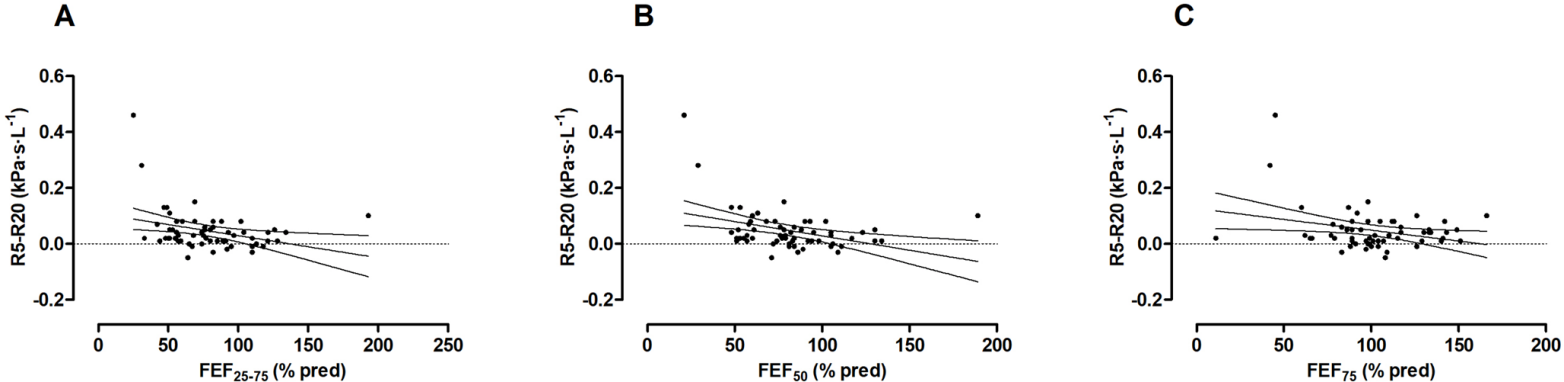
Correlation between R5–R20 (kPa·s·L⁻¹) and spirometric indices of small airway function in 60 AATD subjects: FEF25–75% (A), FEF50% (B), and FEF75% (C). Each point represents one subject; solid lines show the regression with 95% CI

The relationship between spirometry and IOS parameters of SAD were reported in Table 3; Fig. 2.

**Table 3:** Correlation between Spirometry and IOS parameters in 60 AATD subjects with effect size interpretation (low effect size for r value around 0.1, medium for r around 0.3, and large for r more than 0.5)

	FEF_25−75_, % pred	FEF_50_, % pred	FEF_25_, % pred
R5-R20, kPa·s·L − 1	*r* = -0.3199 *p* = 0.0135	*r* = -0.3810 *p* = 0.0029	*r* = -0.2833 *p* = 0.0297
X5, kPa·s·L − 1	*r* = 0.3467 *p* = 0.0071	*r* = 0.3910 *p* = 0.0022	*r* = 0.2216 *p* = 0.0917
AX, kPa·s·L − 1	*r* = -0.3471 *p* = 0.0071	*r* = -0.4115 *p* = 0.0012	*r* = -0.2989 *p* = 0.0215

*FEF*_25−75_ forced expiratory flow between 25 and 75%; *FEF*_50_ forced expiratory flow at 50%; *FEF*_75_ forced expiratory flow at 75%; X5, 5 Hz reactance; AX, reactance area

Fourteen (23%) out of 60 subjects had at least two of three forced expiratory flows, FEF_25−75_, FEF_50_, and FEF_75_, less than 65% of predicted values (FEF+). Furthermore, 6 (10%) out of 60 individuals had a R5-R20 value greater than 0.07 kPa·s·L − 1 (R5-R20+) and 8 (13%) met both criteria.

When we considered patients with asthma or bronchiectasis (*n* = 31), SAD was found in 20 individuals (65%). Specifically, 11 patients (36%) had SAD according to spirometry criteria, 4 (13%) according to IOS criteria, and 5 (16%) according to both. In relatives (*n* = 29), 8 (28%) showed evidence of SAD: 3 (10%) by spirometry, 2 (7%) by IOS, and 3 (10%) by both criteria. No significant difference was found between the percentage of SAD patients with asthma or bronchiectasis, as related to the percentage of SAD of the relatives. (χ^2^ = 3.060, *p* = 0.08)

In smoking subjects (*n* = 20), SAD was found in 8 individuals (40%) of which 5 patients; 4 patients (20%) according to spirometry, and 4 patients (20%) according to both spirometry and IOS criteria. In non-smoking subjects (*n* = 40), SAD was found in 20 individuals (50%), including 15 patients, 10 patients (25%) according to spirometry, 6 (15%) by IOS criteria and 4 by both criteria (10%) (χ^2^ = 0.199, *p* = 0.65).

The relative strength of agreement between FEF + and R5-R20 + was slight, being the κ value 0.22.

Eight out of 60 subjects (13%) reported dyspnea with an mMRC score ≥ 2. Individuals with mMRC score ≥ 2 had R5-R20 values significantly greater than subjects with mMRC < 2. In addition, in all subjects, a significant correlation was found between mMRC and R5-R20 values (*r* = 0.38, *p* = 0.003). Moreover, when comparing the percentage of R5-R20 + between subjects with mMRC ≥ 2 and those with mMRC < 2, we found a significant difference (50% vs. 19%; χ2 = 3.66, *p* = 0.05). No difference was found when comparing FEV+ across mMRC values.

## Discussion

In the present study, we investigated the function of small airways in 60 non-COPD-AATD subjects. The 52% of patients had a diagnosis of asthma and/or bronchiectasis, and the remaining 48% were referred to our outpatient clinic as relatives of AATD patients. SAD was diagnosed in 23% of subjects through FEF25-75, FEF50, and FEF75 criteria; in 10% through R5-R20, and in 13% through both criteria. A slight agreement was found between spirometry and IOS criteria These findings support IOS as a partially complementary tool for the early detection of peripheral airway impairment in AATD. Furthermore, it is of note that IOS but not spirometry criteria were related to breathlessness perception in AATD patients without airflow obstruction, with IOS proving to be a clinically more sensitive measure.

Although spirometry and oscillometry parameters were significantly related, our findings revealed a lack of agreement between the two techniques (k = 0.22), suggesting that they provide different but partially complementary information on small airway impairment. IOS provides an effort-independent measurement of respiratory impedance during quiet tidal breathing, whereas spirometry is effort-dependent and influenced by lung elastic recoil, airway closure, and patient cooperation. We also found a significant association between IOS-defined SAD and clinical symptoms, as measured by the mMRC dyspnoea scale. By contrast, no relationship was found when SAD was defined using spirometry criteria alone.

Our findings are in line with those by Ferrari da Cruz et al. (2021), who described three AATD patients with preserved spirometry but significant alterations in nitrogen washout tests, suggesting early involvement of the small airways despite the absence of clinical symptoms or pulmonary function abnormalities. Unlike our cohort, which applied IOS and spirometric parameters to detect SAD, their findings were limited to case reports without correlation to respiratory symptoms.

Similarly, Stockley et al. (2017) investigated the value of SAD in a wide cohort of 196 AATD patients. The authors used a cut-off of 80% of FEF25-75 predicted value to select AATD patients with SAD. Notably, in a subgroup consisting in 40 AATD patients without COPD and SAD, the reduced value of FEF25-75 was associated with worse health status and a more rapid decline in FEV₁ over time (Stockley et al. 2017). It is of note that the literature about the use of FEF25-75 value to detect SAD is inconclusive. In a large adult cohort, FEF25–75 was found to provide no additional clinical value beyond what was already offered by FEV₁, FVC, and the FEV₁/FVC ratio (Quanjer et al. 2014). Moreover, this parameter showed no association with small airway inflammation (Sutherland et al. 2004).

In absence of a gold standard functional criteria to assess SAD, we used more strict spirometry criteria, including FEF25-75, FEF50 and FEF75, by choosing a lower cut-off (Xiao et al. 2020). Furthermore, we used IOS measurements to detect SAD, since R5-R20 was found more sensitive than spirometry to intercept peripheral airway dysfunction (Pisi et al. 2023) and we found a percentage of SAD higher by spirometry than IOS. Interestingly, although limited by sample size, the finding that the prevalence of SAD was not different in patients with asthma, bronchiectasis, or a history of smoking, as compared to those without these conditions, reinforces the hypothesis of a strict relationship between AATD and SAD. Notably, SAD was also detected in relatives without respiratory disease, suggesting that early peripheral airway involvement may occur even in clinically asymptomatic individuals. This supports SAD as marker of susceptibility within the AATD spectrum, preceding functional impairment.

The pathophysiological implications of the impairment of small airways in AATD patients are further supported by the review of Toumpanakis et al. (2023), who highlighted the central role of small airways in the natural history of patients with AATD. The authors underlines that structural alterations such as loss of alveolar attachments, shortening and narrowing of preterminal bronchioles occur early in AATD and may precede emphysema formation. These changes can contribute to peripheral airway resistance and ventilation inhomogeneity, both of which are detectable by IOS parameters.

To our knowledge, this is the first study assessing the agreements between spirometry and IOS in AATD individuals. Previous studies have also reported poor agreement between these methods but in other respiratory conditions. Lu et al. (2022), found a similar result in a large cohort of COPD patients. Likewise, in asthmatics our previous study demonstrated slight concordance between spirometry and IOS criteria for SAD detection (Pisi et al. 2023). This finding may be explained by the different physiological mechanisms of the two techniques: spirometry relies on forced expiration and is influenced by lung elastic recoil and patient effort, whereas IOS assess respiratory impedance during quiet tidal breathing, which is more physiological and effort independent. Moreover, in the same study, IOS but not spirometry was significantly associated with respiratory symptom control.

Similarly, in the present study, IOS, but not spirometry defined SAD, showed a significant association with respiratory symptoms, such as dyspnoea measured by the mMRC scale. These findings further reinforce the concept that IOS may be more sensitive than spirometry in detecting early alterations in small airways.

This study has some limitations. First, the single center retrospective design may have introduced selection bias and may limit the generalizability of the findings. Second, the sample size was relatively small. Third, because of the genetic heterogeneity of our cohort, findings cannot be generalized to individuals with severe AATD alone. Finally, we aknowledge that the lack of long-term follow-up limits the assessment of disease progression.

However, the rigorous selection criteria and the biochemical confirmation of AATD through serum AAT measurement contribute to the reliability of the data, and the population included is representative of the broader AATD population. Future prospective, multicenter studies with larger sample sizes and longitudinal follow-up are needed to confirm these findings and to better define the clinical role of IOS in the early detection of SAD in AATD.

## Conclusions

In summary, our study confirms the role of SAD as an early functional marker in AATD patients and their relatives without airflow obstruction. In the absence of a gold standard for diagnosing SAD in this cohort, spirometry and IOS provide different but partially complementary information. Our findings also demonstrated that IOS is a more sensitive tool than spirometry for assessing clinical impairment in AATD subjects.

## Supplementary Information

Below is the link to the electronic supplementary material.

Supplementary Material 1
